# Zoonotic Malaria – Global Overview and Research and Policy Needs

**DOI:** 10.3389/fpubh.2014.00123

**Published:** 2014-08-18

**Authors:** Ranjan Ramasamy

**Affiliations:** ^1^Faculty of Infectious and Tropical Diseases, London School of Hygiene and Tropical Medicine, London, UK

**Keywords:** African apes, *Anopheles* vectors, human malaria, malaria control, malaria transmission, non-human primate malaria, *Plasmodium*, zoonosis

## Abstract

The four main *Plasmodium* species that cause human malaria, *Plasmodium falciparum*, *Plasmodium vivax*, *Plasmodium malariae*, and *Plasmodium ovale*, are transmitted between humans by mosquito vectors belonging to the genus *Anopheles*. It has recently become evident that *Plasmodium knowlesi*, a parasite that typically infects forest macaque monkeys, can be transmitted by anophelines to cause malaria in humans in Southeast Asia. *Plasmodium knowlesi* infections are frequently misdiagnosed microscopically as *P. malariae*. Direct human to human transmission of *P. knowlesi* by anophelines has not yet been established to occur in nature. Knowlesi malaria must therefore be presently considered a zoonotic disease. Polymerase chain reaction is now the definitive method for differentiating *P. knowlesi* from *P. malariae* and other human malaria parasites. The origin of *P. falciparum* and *P. vivax* in African apes are examples of ancient zoonoses that may be continuing at the present time with at least *P. vivax*, and possibly *P. malariae* and *P. ovale*. Other non-human primate malaria species, e.g., *Plasmodium cynomolgi* in Southeast Asia and *Plasmodium brasilianum* and *Plasmodium simium* in South America, can be transmitted to humans by mosquito vectors further emphasizing the potential for continuing zoonoses. The potential for zoonosis is influenced by human habitation and behavior as well as the adaptive capabilities of parasites and vectors. There is insufficient knowledge of the bionomics of *Anopheles* vector populations relevant to the cross-species transfer of malaria parasites and the real extent of malaria zoonoses. Appropriate strategies, based on more research, need to be developed for the prevention, diagnosis, and treatment of zoonotic malaria.

## Introduction

The history of the many discoveries made over a period of more than 100 years showing that human malaria was caused by a protozoan parasite that is transmitted by mosquito vectors has been extensively documented [Ref. (1) and references therein]. It is now known that infections with four species of malaria parasites, viz. *Plasmodium falciparum*, *Plasmodium malariae*, *Plasmodium ovale*, and *Plasmodium vivax*, transmitted between humans by mosquito vectors of the genus *Anopheles* are primarily responsible for human malaria. The World Health Organization in its latest malaria report (2) estimated that about 207 million persons had clinical disease and 627,000 died worldwide from malaria in 2012. Human malaria is endemic in many parts of sub-Saharan Africa, South and Southeast Asia, and Central and South America (3, 4). About 3.4 billion people in the world are at risk of malaria, although the risk is relatively low outside Africa and South/Southeast Asia. *P. falciparum*, the dominant parasite in Africa (3), is responsible for 92% of the deaths that occur mostly among children living in sub-Saharan Africa. *P. vivax*, a common human malaria parasite in much of Asia and South America (4), is regarded to cause serious disease but rarely death. However, there are indications that severe vivax malaria and attendant fatalities may have increased in Asia-Pacific and South America over the past 5 years (5). *P. malariae* and *P. ovale* are the two other rarer human malaria parasites.

Approximately 250 species of *Plasmodium* are presently believed to parasitize mammals, birds, and reptiles. All are transmitted by insect vectors. More than 30 species of *Plasmodium* have been reported in non-human primates, including apes, gibbons, and New and Old World monkeys. All primate malarias are believed to be transmitted only by *Anopheles* mosquitoes.

Some monkey malaria parasites have been experimentally transmitted to humans in the past through the bites of infected mosquitoes. They include *Plasmodium cynomolgi* (6–8), *Plasmodium knowlesi* (9, 10), and *Plasmodium inui* (11) from Old World monkeys, *Plasmodium brasilianum* (12) and *Plasmodium simium* (13) from New World monkeys, and *Plasmodium schwetzi* (now regarded to be either *P. vivax* or *P. ovale*-like parasites) from chimpanzees (14). However, recent discoveries, made possible by the use of polymerase chain reaction (PCR) and high throughput DNA sequencing, now establish that human malaria can also be a zoonotic disease.

## *Plasmodium knowlesi* Zoonosis in Asia

Natural human infection with *P. knowlesi*, a common parasite of mainly macaque (*Macaca* species) monkeys in Southeast Asia, has been known to be possible for half a century (15). The more recent discovery of a focus of human infections in the Kapit division of the Sarawak state of Malaysian Borneo, however, showed that *P. knowlesi* zoonosis was more prevalent than previously suspected (16). The infections in Kapit were misdiagnosed by routine microscopy of blood films as being caused mainly by *P. malariae*. This is because the late blood stages of *P. knowlesi* and *P. malariae* are morphologically very similar. The subsequent retrospective use of *P. knowlesi*-specific primers for PCR amplification and DNA sequencing of the genes for the small subunit ribosomal RNA (*ssrRNA*) and circumsporozoite protein (*csp*) was essential for correct species identification in Kapit (16). Human *P. knowlesi* infections were subsequently demonstrated in Pahang in peninsular Malaysia and in Sabah in Malaysian Borneo by species-specific PCR (17). A more recent retrospective *ssrRNA*-based PCR analysis of malaria blood samples showed that human infection with *P. knowlesi* is in fact widespread in peninsular Malaysia as well as the states of Sarawak and Sabah in Malaysian Borneo, accounting for 96% of all malaria cases in one peninsular hospital (18). Erroneous microscopic identification of *P. knowlesi* as a human malaria parasite in blood films was reported to be common in peninsular Malaysia (18) as was the case in Malaysian Borneo (16, 17). Mixed infections of *P. knowlesi* and human malaria parasites in patients have been observed in both peninsular Malaysia and Malaysian Borneo (17, 18). The use of DNA-based identification methods has since documented human *P. knowlesi* infections in much of Southeast Asia, ranging from the Andaman and Nicobar islands of India in the West to the Philippines in the East [Ref. (19) and references therein].

*Plasmodium knowlesi* is normally a parasite of *Macaca fascicularis* (the long-tailed or crab-eating macaque), *Macaca nemestrina* (the pig-tailed macaque), *Trachypithecus obscuras* (dusky leaf monkey or spectacled langur), and *Presbytis melalophus* (banded leaf monkey or brown langur) [Ref. (19) and references therein]. The main documented vectors are members of the *Anopheles leucosphyrus* group, some of which have been reported to feed equally well on humans and monkeys [Ref. (19) and references therein, Ref. (20)]. Within the leucosphyrus group, *Anopheles balabacensis* is a particularly efficient vector of simian *P. knowlesi* in Malaysia as is *Anopheles dirus* in Vietnam [reviewed in Ref. (9, 20, 21)]. A map of the likely range of *P. knowlesi* based on the distribution of host monkey species and the relevant anopheline vectors, suggests that a potential for *P. knowlesi* zoonosis exists across large tracts of the populous South and Southeast Asian region (19). Because microscopy and rapid diagnostic tests commonly employed to diagnose malaria lack the requisite specificity, it is possible that many more patients with *P. knowlesi* malaria may have been unknowingly misdiagnosed in South and South East Asia.

Experimental *P. knowlesi* inoculations have been used in the past to treat neurosyphilis (9). The parasite has a 24 h asexual cycle in blood and therefore produces a quotidian fever that can potentially differentiate it from the 72 h quartan fever of *P. malariae* infections in humans. However, the clinical symptoms of naturally acquired *P. knowlesi* infections in humans are often not very specific and the infections can sometimes prove severe and fatal with high parasitemias [Ref. (9) and references therein]. Mixed infections of *P. knowlesi* with human malaria parasites are not uncommon in patients (17, 18, 21) and human infection with *P. knowlesi* in some sites in Vietnam has been reported to be asymptomatic (21). This contrasts with the milder symptoms and lower parasitemias observed with *P. knowlesi* in its natural monkey hosts (9). More extensive investigations on the clinical features and treatments of *P. knowlesi* infections in humans are warranted by the increasing realization that such infections are more common than previously reported in countries like Malaysia (18). From the treatment point of view, it is relevant to note that *P. knowlesi* strains from patients in Malaysian Borneo were reported to be sensitive to artemisinins, moderately but variably sensitive to chloroquine, and less sensitive to mefloquine (22). It is not clear whether the less than desirable sensitivity of *P. knowlesi* to chloroquine and mefloquine is a result of drug selection in humans and/or the intrinsic biology of the parasite, and this merits further investigation.

Despite the experimental transfer of *P. knowlesi* infections to humans and documented natural infections in Southeast Asia, the direct transfer of *P. knowlesi* from one infected person to another uninfected human by mosquito vectors has yet to be unequivocally demonstrated to occur in nature. Rather, human infections appear to be acquired from monkeys when people venture into forests. Epidemics of *P. knowlesi* malaria have not been reported and clustering of infections was not observed at Kapit, Sarawak in Malaysian Borneo (16). However, *P. knowlesi* is able to form mature gametocytes in the blood of infected persons (23, 24). *P. knowlesi* sporozoites are also detectable in the salivary glands of *An. dirus*, a human malaria vector in Vietnam, commonly as mixed infections with *P. vivax* and *P. falciparum* sporozoites (21). The existing data therefore raise the possibility that human to human transmission of *P. knowlesi* may occur in areas with a high density of *P. knowlesi* zoonosis. However, there is presently no evidence to firmly establish that such transmission takes place, and if it does occur, the possible rates of knowlesi malaria transmission between humans and its epidemiological significance need to be investigated.

## *Plasmodium cynomolgi* Zoonosis in Asia

The experimental infection of humans with *P. cynomolgi*, commonly a parasite of macaque monkeys in Asia, through the bites of infected *Anopheles freeborni* mosquitoes was demonstrated half a century ago (6). However, the first natural infection of a human was only reported very recently in peninsular Malaysia (25). Microscopic examination of the blood film from this patient suggested infection with *P. vivax*. *P. cynomolgi* blood stages are morphologically very similar to those of *P. vivax*. A common nested PCR test (26) yielded an amplified fragment of *ssrRNA* whose size was characteristic of *P. vivax*. A more discriminating PCR followed by DNA sequencing of *ssrRNA*, however, showed conclusively that the parasite causing the infection was in fact *P. cynomolgi* (25). The clinical presentation of this patient was mild with 24 h cyclic chills rather than the expected tertian fever resulting from the 48 h asexual blood stage cycle of *P. cynomolgi*. The patient lived adjacent to a small forest where macaques were present. The suspected mosquito vector in this case was *Anopheles cracens*, belonging to the *An. leucosphyrus* group of mosquitoes, and a known vector of the simian malaria parasites *P. cynomolgi* and *P. inui* (25). This case suggests that other instances of human infections with *P. cynomolgi* in South and Southeast Asia may have been similarly misdiagnosed as *P. vivax* infections by microscopy.

## *Plasmodium simium* and *Plasmodium brasilianum* as Zoonoses in the New World

*Plasmodium simium* is a parasite of the platyrrhine monkeys of South America. The New World platyrrhine monkeys diverged from catarrhine monkeys of the Old World about 30–35 million years ago (27). Sequence analysis of genes coding for the merozoite surface protein-1 (*msp1*) and cytochrome *b* (*cytb*) and the *ssrRNA* and *csp* genes from worldwide isolates of *P. vivax* and a limited number of *P. simium* isolates from South America showed that the two parasites were very closely related (28). It is unlikely that *P. vivax* originated in the New World from *P. simium* because there is now evidence to show that *P. vivax* in humans arose from related parasites in African apes (29). This conclusion is supported by the greater genetic diversity of *P. vivax* worldwide compared to *P. simium*, which is found only in South America (28). It is therefore more likely that *P. simium* arose from *P. vivax* that was transferred to platyrrhine monkeys by infections prevalent in settlers from the Old World in the post-Columbus era. At least two such cross-species transfers must have taken place as two different alleles of the *P. vivax csp* are also found in *P. simium* (28).

Natural infection of a human by *P. simium* was reported from Brazil in 1966 (13). A volunteer collecting mosquitoes by human landing catches in a forest became infected with the parasite, which was identified by careful microscopic examination of blood films to be *P. simium*. The infection could be syringe-transferred from his blood to a platyrrhine squirrel monkey (*Saimiri* spp.) It was suspected that platyrrhine howler monkeys (*Alouatta fusca*) were the original source of the infection, and *Anopheles cruzi* the vector, in this instance of zoonosis. It was suggested that human *P. simium* infections contracted in forests may often be misdiagnosed as *P. vivax* during routine microscopy due to the presence of similar patterns of stippling or Schuffner’s dots in infected red blood cells, and because many microscopists might be unaware of the possibility of simian malaria parasites infecting humans (13).

*Plasmodium brasilianum* is a parasite of many species of platyrrhine monkeys in South and Central America. Sequence analysis of *csp*, *msp1*, and *ssrRNA* of *P. malariae* and *P. brasilianum* showed that the two parasites were very closely related but the likely direction of a cross-species transfer could not be established due to the limited number of parasite isolate sequences available for analysis (28). There is now evidence to suggest that parasites closely related to *P. malariae* are found in African apes (30, 31). If it can be shown that *P. malariae* in man, like *P. falciparum* (30) and *P. vivax* (29), also arose through cross-species transfer from African apes in Africa, it would appear plausible that *P. brasilianum* in platyrrhines is a result of the cross-species transfer of *P. malariae* brought to the New World by settlers in the post-Columbus era.

*Anopheles freeborni* mosquitoes infected by feeding on a platyrrhine spider monkey, *Ateles geoffroyi geoffroyi*, from Panama carrying *P. brasilianum*, have been shown to transmit the parasite through biting to five human volunteers (12). This raises the possibility that the transmission of *P. brasilianum* from platyrrhine monkey hosts to humans occurs naturally in South and Central America with the resulting human infections being diagnosed as *P. malariae* by microscopy and other diagnostic tests. Since very few gene sequences are available for the two species, and the existing ones show little difference between them, establishing zoonosis through DNA-based diagnostics is difficult at the present time. It also seems likely that *P. malariae* and *P. brasilianum* are in fact variants of the same parasite species that are able to infect both humans and platyrrhine monkeys in the New World.

## *Plasmodium falciparum* as an Ancient Zoonosis in Africa

It was believed for some time, based on limited DNA sequencing, that the closest relative to human *P. falciparum* was the chimpanzee (*Pan troglodytes*) parasite *Plasmodium reichenowi* (32). The origin of *P. falciparum* was, however, recently established through sequencing a number of genes from parasite DNA isolated from fecal samples of wild apes in Africa (30). DNA sequence analysis showed that parasites more closely related to *P. falciparum* than *P. reichenowi* are prevalent in western gorillas (*Gorilla gorilla*) in West and Central African forests. Analysis of the sequences of the mitochondrial *cytb* showed that the *P. falciparum*-related ape parasites could be phylogenetically classified into six host-specific clades (30) tentatively named as *P. reichenowi*, *Plasmodium gaboni*, and *Plasmodium billicollinsi* in chimpanzees and *Plasmodium praefalciparum*, *Plasmodium adleri*, and *Plasmodium blacklocki* in western gorillas (33). All known human *P. falciparum cytb* sequences formed a distinct lineage within the gorilla-specific *P. praefalciparum* clade (30). Analysis of the apicoplast caseinolytic protease C (*clpC*) and nuclear lactate dehydrogenase (*ldh*) genes, and the entire mitochondrial genome, yielded a phylogenetic relationship compatible to that obtained with *cytb*. Therefore, *P. falciparum* most probably arose through the cross-species transfer of an ancestral *P. praefalciparum*-like parasite from gorillas that then successfully adapted itself to humans (30). *Anopheles moucheti*, a known human malaria vector that is found in forested areas of Central Africa, has been shown to carry *P. praefalciparum* in Gabon (34). Inoculation of *P. praefalciparum* into humans is therefore very likely to be a continuing process in West and Central Africa but productive infections in potentially exposed humans in Cameroon were not detected and are therefore likely to be extremely rare (35). However, more studies involving other African sites are needed to further clarify this aspect.

Apes are not readily infected with *P. falciparum* and in order to obtain high parasitemia, chimpanzees have to be splenectomized (36). Platyrrhine owl (*Aotus* spp.) and squirrel (*Saimiri* spp.) monkeys are also infectible with *P. falciparum*, but a period of adaptation in splenectomized animals is required (36). It has been proposed that the adaptation and host specificity of *P. falciparum* for humans is a result of the specific interaction of a *P. falciparum* protein ligand PfEBA175 with *N*-acetylneuraminic acid on human erythrocyte glycophorin A (37). However, more recent evidence suggests that the specificity may be due to the interaction between PfRh5, an essential erythrocyte invasion ligand found in the rhoptry organelles of the parasite, and the corresponding human erythrocyte receptor termed basigin (38). Basigin or CD 147 is responsible for the Ok blood group phenotype. PfRh5 does not bind gorilla basigin and only binds weakly to chimpanzee basigin and these properties have been suggested to cause the host restriction of *P. falciparum* to humans (38).

Analysis of single nucleotide polymorphisms (SNPs) in two *P. falciparum* genes for common housekeeping proteins (P type Ca^2+^-ATPase, *serca*, and adenylosuccinate lyase, *adsl*), from parasite isolates in different parts of the Old World showed that genetic diversity in *P. falciparum* decreased significantly with the distance from sub-Saharan Africa (39). This finding may be interpreted as being compatible with a hypothesis that the carriage of *P. falciparum* from an origin in sub-Saharan Africa to the Old World was a result of the migration of modern humans (*Homo sapiens*) and/or ancestral *Homo* species to whom *P. falciparum* had become adapted.

## *Plasmodium vivax* as an Ancient and Continuing Zoonosis in Africa

Sequence analysis of mitochondrial DNA from ape fecal and monkey blood samples in tropical sub-Saharan African forests also show that *Plasmodium* parasites genetically very similar to human *P. vivax* are common in wild chimpanzees (*P. troglodytes*) and western (*G. gorilla*) and eastern gorillas (*Gorilla beringei*) but not bonobos (*Pan paniscus*) or monkeys (29). Human *P. vivax* sequences from different parts of the world formed a distinct lineage within the ape sequences but the data suggest that the ape and human parasites belong to a single species, *P. vivax*. Analysis of the apicoplast *clpC* and four nuclear genes (*ldh*, *asl* coding for adenylosuccinate lyase, β-*tub* coding for β tubulin and *crk-2* coding for cell division cycle 2-related kinase) also produced results that were consistent with this conclusion (29).

*Plasmodium vivax* uses the Duffy blood group antigen molecule on red blood cells as the main receptor for binding and subsequent invasion. There are three major alleles for the antigen, viz. FY*A and FY*B, which differ by a single amino acid, and FY*O that gives rise to the absence of the receptor and the Fy(a-b-) serological phenotype in homozygous individuals. The Fy(a-b-) phenotype has been commonly associated with near complete resistance to *P. vivax* in most of Africa (40). The FY*O allele is estimated to have been selected in Africa approximately 33,000 years ago, i.e., after the initial migration of *H. sapiens* out of Africa, probably because of the protection provided against vivax malaria (41). However, approximately 5% of West Africans and a greater proportion of East Africans remain Duffy antigen positive and there is also evidence that *P. vivax* has evolved to use receptors other than the Duffy blood group antigen in East and West Africa (42–44).

The greater similarity of human *P. vivax* to ape *P. vivax* than human *P. falciparum* to gorilla *P. praefalciparum*, suggests that there may be continuing cross-species exchange of *P. vivax* between humans and apes in tropical Africa. Zoonosis was recently confirmed by sequence analysis of parasite DNA from a Caucasian European traveler, who contracted *P. vivax*-like malaria while working in the Central African Republic, which showed that the parasite had SNPs characteristic of ape and not human *P. vivax* (45). *P. vivax* with SNPs reportedly characteristic of human *P. vivax* has been identified in African apes confirming that the reverse transfer is also possible (45). *Anopheles moucheti* and *Anopheles vinckei* have been shown to carry ape-like *P. vivax* in Gabon and are potential vectors for zoonotic transmission to humans in or near forests (34, 45). The Fy(a-b-) phenotype or the absence of the Duffy antigen in a majority of the West and Central Africans would, however, protect against zoonotic *P. vivax* but such protection will not apply to the minority of Duffy antigen positive Africans and non-native populations or infection with *P. vivax* strains that can use alternative red blood cell receptors for invasion. Recent evidence suggests that Duffy antigen negative persons in Congo have antibodies specific for *P. vivax* asexual blood stage antigens (46) and that blood stage *P. vivax* infections can be detected by PCR in Duffy antigen positive and negative asymptomatic individuals in Cameroon (47). These recent observations are consistent with a proposal that Duffy antigen positive apes are a reservoir of *P. vivax* in West and Central Africa for transmission to humans to produce mild infections and elicit antibodies (48). More studies are clearly needed to determine the nature and extent of *P. vivax* zoonosis in Africa.

## *Plasmodium malariae* and *Plasmodium ovale* as Possible Ancient and Continuing Zoonoses in Africa

Studies in the 1940s by Rhodain suggested that a chimpanzee (*P. troglodytes*) parasite *Plasmodium rhodaini* was very similar, if not identical, to the human quartan malaria parasite *P. malariae*, with both parasites being readily transferable between humans and chimpanzees [reviewed in Ref. (49)]. Analysis of a limited number of *cytb* and *ssrRNA* sequences available now suggests that both *P. malariae* and *P. ovale* have closely related counterparts in wild chimpanzees in tropical Africa (30, 31). A larger number of *P. malariae* and *P. ovale* and ape sequences are needed for detailed analyses for determining the likely ancestral parasites of the two human species. Presently available data are, however, compatible with a hypothesis that human *P. malariae* and *P. ovale*, like *P. falciparum* and *P. vivax*, originated by cross-species transfer from African apes and then spread worldwide. Testing this hypothesis would require DNA sequences from a greater number of human *P. malariae* and *P. ovale* isolates worldwide and isolates of related parasites from wild African apes.

## Under-Diagnosis of Zoonotic Infections in Humans

The continuing difficulty in correctly identifying *P. knowlesi* in Southeast Asia, because of its similarity to *P. malariae* in blood films as discussed in Section “*Plasmodium knowlesi* Zoonosis in Asia” above, illustrates the potential for under-diagnosis of other forms of zoonotic malaria in many parts of the world. Microscopists working in hospitals and health centers in malaria-endemic countries where zoonoses may occur are not likely to differentiate between human parasites and closely related zoonotic parasites when routinely screening blood films. Antibody-based rapid diagnostic kits suitable for detecting *P. knowlesi* in blood with acceptable specificity and sensitivity are not presently available (50). It has also been suggested that presumptive drug treatment of malaria-like symptoms, and the lack of resistance to common anti-malarial drugs in *P. praefalciparum*, can rapidly cure zoonotic infections so that such infections may therefore go undetected in sub-Saharan Africa (33). Zoonotic malaria species may also develop low parasitemias in humans, so that their presence may be masked by coinfection with more virulent human species like *P. falciparum*. This has been observed for *P. knowlesi* in Vietnam (21). The availability of SNPs specific for ape parasites (e.g., for *P. praefalciparum* and ape *P. vivax*) will facilitate identification of zoonotic infections as already demonstrated for *P. vivax* (45). However, ape malarial parasites related to *P. falciparum* including *P. praefalciparum* were not detected in humans who are likely to have been inoculated with such parasites by mosquito vectors in remote rural areas of Cameroon (35). Such DNA-based tests are not available for routine identification of zoonotic malaria parasites in malaria-endemic countries. They have also not yet been developed for *P. malariae* and *P. ovale* in Africa and may not be applicable in South America for differentiating *P. simium* from *P. vivax* and *P. brasilianum* from *P. malariae*.

Of the many *Plasmodium* species known to parasitize non-human primates, only a few species have been shown to be naturally transmitted to humans at the present time (Table 1). Although experimentally transmissible to humans (11), natural human infection with *P. inui* has not yet been documented. The absence of suitable diagnostic methods may explain the failure to detect *P. inui*, and perhaps many other non-human primate malaria parasite species, in humans. However, there are several possible causes that can prevent such other non-human primate *Plasmodium* species from naturally infecting humans. These include the inability to infect and form sporozoites in mosquito vectors that feed on humans, susceptibility to human innate immune responses, absence of compatible parasite ligands and human erythrocyte and hepatocyte receptors that facilitate productive invasion, and existence of intracellular hurdles that bar development even if host cell invasion were to be successfully achieved.

**Table 1:** **Documented ongoing malaria zoonoses**.

*Plasmodium* species	Location	Reference
*P. knowlesi*	Southeast Asia	(15–21)
*P. cynomolgi*	Malaysia	(25)
*P. vivax*	West Africa	(45)
*P. vivax*/*P. simium*	South America	(13)

## Changes in Human Habitation and Ecology as Well as Adaptive Changes in Parasites and Vectors may Facilitate the Emergence of New Human Malaria Parasites from Non-Human Primates

Increasing populations coupled with a demand for more agricultural land and timber is leading to extensive deforestation in malaria-endemic countries of the tropics. Humans are also venturing into forested areas in greater numbers for mining, lumbering, and recreation, and more people are beginning to live close to forests. Wild ape populations are also diminishing in Africa as are wild monkey populations throughout the world (51). Anopheline vectors that are responsible for sylvatic transmission of malaria parasites are therefore likely to be faced with decreasing numbers of monkey and ape hosts and increasing numbers of potential human hosts. Examples of anopheline malaria vectors becoming more anthropophilic over the course of a few years as a result of a shortage of animal hosts have been long documented (52). Mosquito vectors are generally highly versatile in adapting to environmental changes (53). Therefore, it is likely that ongoing changes in human habitation patterns and ecology as well as vector adaptation will lead to an increasing probability of humans becoming inoculated with monkey and ape malaria parasites and vice versa. In rare cases, such cross-species transfer may result in a non-human primate *Plasmodium* species varying sufficiently to adapt itself to human–human transmission, as was the likely case for the ancestor of *P. falciparum*. There is also the rare possibility that human malaria parasites may undergo genetic recombination with closely related ape and monkey parasites to yield new parasite strains that are more virulent to humans. Malaria parasites are haploid in their primate hosts, and meiosis and zygote formation takes place in the mid gut of mosquitoes. Genetic recombination between different *P. falciparum* strains occurs in mosquitoes (54). A mosquito vector infected with gametocytes of two closely related human and non-human primate parasites at the same time can theoretically serve as a vehicle for similar genetic recombination. Such a mix of gametocytes can be acquired through rapid sequential blood meals on infected humans and non-human primates in forested areas or in the case of *P. vivax* be acquired from a co-infected human or non-human primate. *P. vivax* may be particularly prone to such a recombination process since it appears to be readily able to switch between apes and humans in Africa as discussed in Section “*Plasmodium vivax* as an Ancient and Continuing Zoonosis in Africa” and between platyrrhine monkeys and humans in South America as discussed in Section “*Plasmodium simium* and *Plasmodium brasilianum* as Zoonoses in the New World.” An epidemiologically pertinent but molecularly distant example from another pathogen is the swine influenza variant 2009 A (H1N1) that caused the 2009 influenza pandemic. It contained many genetic re-assortments with common swine influenza virus genes that resulted greater pathogenicity to humans (55). Genetic recombination is, however, likely to occur much less frequently with malaria parasites than influenza viruses because of the much greater complexity associated with the parasite sexual recombination process, but the possibility nevertheless needs to be borne in mind.

## Research and Policy Needs

Not enough is presently known about the bionomics of anopheline vectors that transmit ape and monkey malaria parasites that are closely related to the human ones. This is more so in tropical Africa and South America than Asia. Details of their feeding preference for human and non-human primate hosts, resting habits, dispersal distances after blood feeding, insecticide sensitivities, and ongoing adaptation to ecological changes are essential for evaluating the potential role of such vectors in zoonoses. Conversely, there is a need to determine the extent to which the more anthropophilic vectors of human malaria feed on apes and monkeys and transmit malaria parasites.

There is also a need for more studies on the sensitivities of zoonotic parasites to common anti-malarial drugs used to treat human malaria and the clinical features of the different types of zoonotic malarias in humans.

The importance of investigating possible zoonoses in communities living in close proximity to forests and people venturing into forests needs to be understood by public health personnel responsible for malaria detection and control. They need accessible resources, such as a collaborating laboratory, to identify suspected cases of zoonotic malaria with DNA-based techniques. Development, if possible, of non-radioactive DNA probes and loop-mediated isothermal amplification of DNA (LAMP)-based diagnostic techniques (56) that can be used for detecting zoonotic species in endemic country laboratories will be particularly helpful in this regard.

Zoonoses, particularly the existence of sylvatic reservoirs of *P. vivax* and *P. malariae* in South America and Africa, can compromise malaria control and eradication efforts. This needs to be recognized by the public health authorities responsible for malaria control. In a broader context, the agencies responsible for health and environmental planning in the tropics need to be aware of the present and likely future changes in levels of exposure to zoonotic malaria and develop appropriate mitigating and preventive strategies.

## Conflict of Interest Statement

The author declares that the research was conducted in the absence of any commercial or financial relationships that could be construed as a potential conflict of interest.
